# Alginate-Bentonite-Based Hydrogels Designed to Obtain Controlled-Release Formulations of Dodecyl Acetate

**DOI:** 10.3390/gels9050388

**Published:** 2023-05-06

**Authors:** Francisco Flores-Céspedes, Matilde Villafranca-Sánchez, Manuel Fernández-Pérez

**Affiliations:** Department of Chemistry and Physics, Research Centre CIAIMBITAL, University of Almería, Agrifood Campus of International Excellence (ceiA3), Crta. Sacramento s/n, 04120 Almería, Spain; villafra@ual.es (M.V.-S.); mfernand@ual.es (M.F.-P.)

**Keywords:** alginate, bentonite, dodecyl acetate, controlled release

## Abstract

Dodecyl acetate (DDA), a volatile compound present in insect sex pheromones, was incorporated into alginate-based granules to obtain controlled-release formulations (CRFs). In this research, not only was the effect of adding bentonite to the basic alginate-hydrogel formulation studied, but also that of the encapsulation efficiency on the release rate of DDA in laboratory and field experiments. DDA encapsulation efficiency increased as the alginate/bentonite ratio increased. From the preliminary volatilization experiments, a linear relationship was found between the DDA release percentage and the amount of bentonite present in the alginate CRFs. Laboratory kinetic volatilization experiments showed that the selected alginate-bentonite formulation (DDAB_75_A_10_) exhibited a prolonged DDA release profile. The value of the diffusional exponent obtained from the Ritger and Peppas model (*n* = 0.818) indicated that the release process follows a non-Fickian or anomalous transport mechanism. Field volatilization experiments showed a steady release of DDA over time from the alginate-based hydrogels tested. This result, together with those obtained from the laboratory release experiments, allowed the obtainment of a set of parameters to improve the preparation of alginate-based CRFs for the use of volatile biological molecules, such as DDA, in agricultural biological control programs.

## 1. Introduction

In the last 50 years, hydrogels have gained noticeable recognition, because they can be used for a broad range of purposes, such as regenerative medicine, controlled-release drug-delivery systems, food additives, hygiene products, and agricultural applications, among others [1].

Hydrogels are swelling polymeric structures that absorb great amounts of water without dissolving (they can contain 20 to 40 times their dry weight) [2,3]. Their three-dimensional structures arise through the crosslinking of polymer chains by covalent bonds, hydrogen bonds, Van der Waals interactions, or physical interactions between determined functional groups, such as –OH, –CONH_2_, –SO_3_H, –CONH, and –COOR groups [1].

Depending on their origin, hydrogels can be classified as natural hydrogels, obtained directly without any chemical reaction; synthetic hydrogels, prepared by a crosslinking process; or composite hydrogels, obtained from a natural hydrogel by adding a nanocomposite.

Over the years, numerous ways of obtaining controlled-release formulations (CRFs) for bioactive molecules have been studied. Encapsulation in hydrogels has become a common technique in the production of controlled-release forms [4]. Hydrogels form discrete spherical capsules that serve as solid substrates on which active ingredients are coated or encapsulated. Much effort has been focused on the development of hydrogel microspheres using natural polymers. Since they are derived from natural sources, they do not require organic solvents, are readily available, and are suitable for chemical modifications. The use of alginates, gellant gum, chitosan, and carboxymethylcellulose by the ionotropic gelation technique has been widely used for this purpose [5].

Alginate is a naturally occurring anionic and hydrophilic polysaccharide found mainly in brown algae. Alginate is made from two copolymers, α-L-guluronic acid (G) and β-D-mannuronic acid (M). The mannuronic and guluronic units are 1→4 connected by glycosidic bonds, creating three different polymer segments: homopolymeric MM or GG blocks and alternating MG blocks. The properties of the material will depend on the M:G ratio [6]. A broad range of uses—as a biomaterial and mainly as a pillar matrix or delivery system—are recognized for alginate [7]. The formation of a water-insoluble hydrogel with divalent calcium cations by ionotropic gelation is an easy, gentle, and environmentally friendly process that provides the possibility of encapsulating numerous bioactive substances or nutrients [8,9,10,11]. The alginate granules thus obtained have been shown to be excellent vehicles as controlled-release systems.

Mixing inorganic nanoparticles with natural or synthetic polymers can be considered a promising strategy to produce a new generation of advanced materials. The incorporation of inorganic nanoparticles into polymeric hydrogels can induce network-active features. Many alginate hydrogels have been prepared by integrating particles, as modifying agents, within a polymeric matrix [8,9,12]. The incorporation of these particles into the polymeric structure increases the mechanical strength, improves the stability, and confers elastomeric properties on the composite networks. Furthermore, the incorporation of such particles can result in a greater degree of control over the release rate of the bioactive agent [13].

Consequently, specific characteristics of the macromolecular network can be developed by the crosslinking of inorganic nanoparticles within polymeric hydrogels [8,14].

Most of the inorganic nanoparticles used in the preparation of hybrid hydrogels are clay minerals. Surface interactions between these clay minerals and the polymeric chains culminate in materials that can be useful for purposes such as the removal of contaminants through adsorption processes [15] or as carriers in controlled-release systems [8,12]. In this research, a nanobentonite, a mineral from the group of phyllosilicates, was used. This nanoclay was selected given its availability, its structural properties, as well as its high cation exchange capacity and large specific surface area [16,17].

The regulations and measures of the European Union regarding agriculture and the sustainable use of phytosanitary products are highly restrictive in relation to the application of synthetic pesticides. This fact, together with the growing resistance shown by pests to treatment with these substances, entails the need to implement effective and safe alternatives for pest control. In an integrated pest management plan, the use of interference methods for population sampling, mass trapping, and sexual confusion using semiochemical substances is becoming increasingly important.

Pheromones, substances with semiochemical activity, and some natural insecticides of plant origin are considered as potential agents for the protection of crops and areas of public interest because they generally have a low environmental impact and low toxicity for mammals [18,19,20]. The use of sex pheromones represents, due to their sensitivity, specificity, and non-toxicity, an alternative to the use of synthetic insecticides for pest control. However, most of the components of pheromones are volatile substances that evaporate quickly in conventional formulations, which limits their use in agriculture [21,22]. The biological activity of these substances will be enhanced if they are properly encapsulated to prevent rapid degradation and at the same time are released into the environment in controlled doses and over controlled time periods. Dodecyl acetate (DDA), an acetate ester, one of the components of insect sex pheromones, was used in this work as a bioactive semiochemical model.

The development of new alginate-based controlled-release formulations to encapsulate DDA was the main purpose of this research. A nanobentonite was used as a modifying agent to control the volatilization/release rate of DDA, not only in the laboratory but also under field conditions.

To achieve this objective, the following procedures were carried out: (i) preparation and optimization of formulations based on DDA/alginate/bentonite; (ii) characterization of the prepared formulations by TGA, DSC, SEM, and FTIR studies; and (iii) evaluation of DDA CRFs through release kinetics studies, both in laboratory and in field experiments.

## 2. Results and Discussion

The optimization of alginate bead formulations was carried out considering DDA encapsulation efficiency and release capacity as the main deciding factors. The parameters that play a role in the alginate gelation process and in the controlled release of DDA were modified according to various experimental designs (Table 1) to determine their influence on the amount of encapsulated semiochemical and on the rate of DDA release from the beads. To help in the study of bead formulation optimization, preliminary tests were performed to characterize the DDA formulations in terms of morphological characteristics and polymeric network arrangements.

Finally, for the optimized formulation, a release-rate study was carried out under controlled laboratory and field conditions. Technical-grade DDA formulations were tested under the same conditions as the optimized formulation to determine the efficiency of alginate beads with respect to controlling the rate of DDA release.

### 2.1. Granule Characterizations

Practical dodecyl acetate loading and encapsulation efficiency (EE) values were calculated using Equations (1) and (2). These results are shown in Table 2.

The encapsulation efficiency of the DDA formulations ranged from 33.79% for the DDAB_80_A_5_ formulation to 80.31% for DDAA_85_. As could be inferred from these values, an increase in the amount of bentonite in the formulation led to a decrease in the encapsulation efficiency. Furthermore, an increase in EE was observed as the alginate content increased. The presence of bentonite in the alginate network results in less crosslinking, resulting in lower DDA entrapment capacity and therefore lower encapsulation efficiency values. The recovery of solids was improved with the addition of bentonite to the alginate formulation, obtaining values higher than 75% (77.15% for DDAB_80_A_5_ and 90.11% for DDAB_65_A_20_)

**Figure 1:**
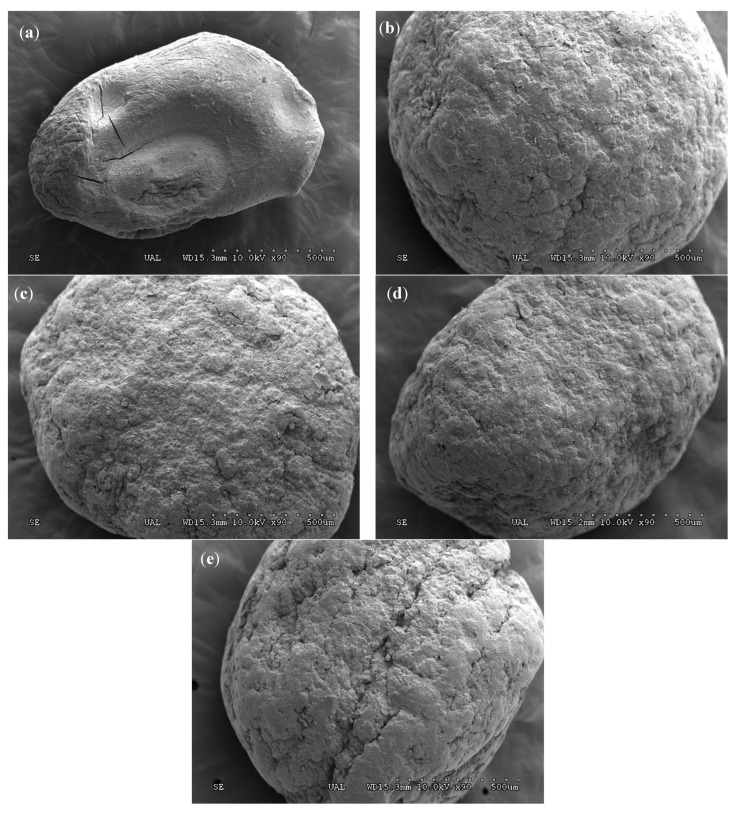
SEM images of the shapes and external morphologies of the CRFs (magnification ×90): DDAA_85_ (**a**), DDAB_65_A_20_ (**b**), DDAB_70_A_15_ (**c**), DDAB_75_A_10_ (**d**), and DDAB_80_A_5_ (**e**).

SEM was used to characterize the network structures of polymeric granules. The external surfaces of the dry beads prepared with five kinds of formulations are shown in Figure 1. They have regularly spherical shapes and smooth surfaces without clear pore structures. In contrast to the DDAA_85_ alginate formulation, the degree of surface roughness of the alginate-bentonite formulations increases as the bentonite content increases. SEM pictures also showed a clearly lower size for DDAA_85_ compared to those prepared by adding bentonite. The sizes of the beads depended on the sizes of the droplets, and the sodium alginate beads were the smallest, though their sizes increased significantly when bentonite was added [23].

**Figure 2:**
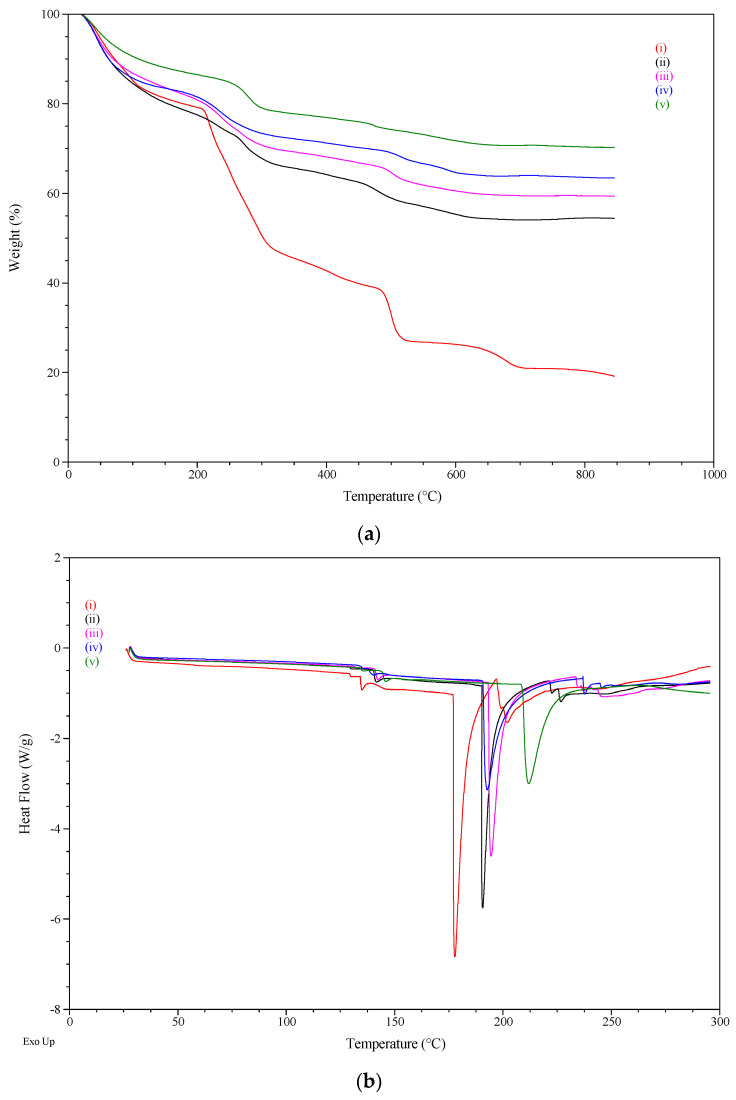
TGA curves (**a**) and DSC curves (**b**): DDAA_85_ (i), DDAB_65_A_20_ (ii), DDAB_70_A_15_ (iii), DDAB_75_A_10_ (iv), and DDAB_80_A_5_ (v).

TGA studies (Figure 2a) showed the higher thermal stability of the formulations containing bentonite compared to the formulation containing only alginate. The formulation prepared with the highest bentonite content (DDAB_85_A_5_) resulted in the low weight loss in the TGA curves, with the highest residue being observed for this formulation (Table 3). This observation coincides with those reported by other authors, who pointed out that, due to the high thermal stability of mineral clays, their introduction into organic materials can improve their thermal stability [24,25,26,27]. 

As shown in the figure, the thermal degradation process of the formulations occurs in two or more stages depending on the complexity of the matrix formed. In the first stage, the temperature ranges between 20 and 150 °C, which corresponded to the loss of absorbed water and bound water interacting with sodium alginate [23]; in a second stage (150–350 °C), the thermal degradation process of the alginate begins, with a weight loss equivalent to 40% of the alginate present in the sample, resulting in the formation of CO, CO_2_, and H_2_O, as well as the evaporation of the active ingredient. The final stage of degradation occurs at temperatures above 350 °C; during this phase, the degradation of the alginate is completed and may correspond to the formation of carbonate, which ensures that the compound has a high thermal stability [28].

Crosslinked calcium matrices were thermally analyzed by differential scanning calorimetry (DSC), as shown in Figure 2b. The DSC thermogram of the Ca-crosslinked DDA-alginate matrix (DDAA_85_) illustrates the appearance of a sharp endothermic band at 178 °C, which probably corresponds to enthalpies of cleavage (i.e., breaking) of the calcium-carboxylate bonds within the complex. Such a sharp endothermic band indicates a highly ordered molecular arrangement (crystallite) that forms a so-called ‘‘egg-box’’ structure within calcium alginate [29].

The effect of bentonite incorporation into the matrix is also shown in Figure 2b. Interestingly, the addition of bentonite led to an upward shift of the 178 °C endothermic band. This band appeared at temperatures from 190 °C to 211 °C, as the amount of bentonite increased. Such an upward shift indicates that bentonite may assist in the formation of stronger Ca-carboxylate coordinate bonds (with higher melting enthalpies). Furthermore, the Ca-alginate-bentonite trait exhibits the same dehydration endothermic peak at approximately 140 °C. 

To verify the presence of DDA molecules incorporated into the alginate formulations, the samples were characterized by infrared spectroscopy. Due to the low concentrations of the incorporated compounds, the FTIR spectra of the samples in conventional KBr disks are strongly dominated by the spectra of the pristine materials (calcium alginate and bentonite).

**Figure 3:**
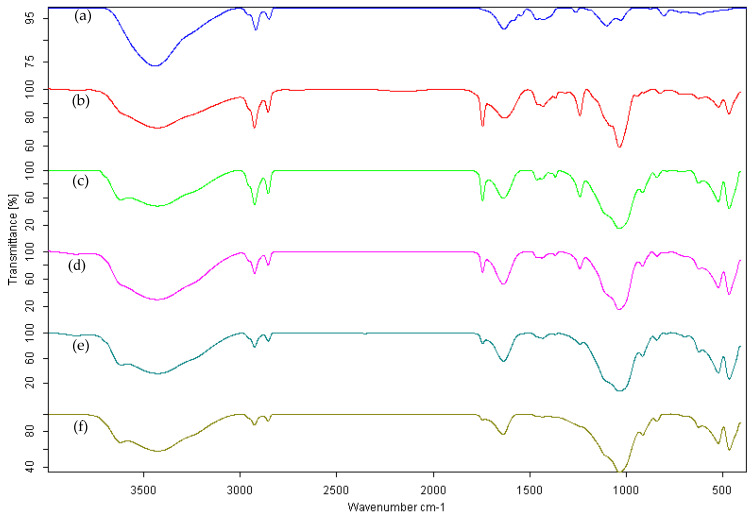
FT-IR spectra of calcium alginate (**a**), DDAA_85_ (**b**), DDAB_65_A_20_ (**c**), DDAB_70_A_15_ (**d**), DDAB_75_A_10_ (**e**), and DDAB_80_A_5_ (**f**).

The FTIR spectra of the calcium alginate and dodecyl acetate-bentonite-alginate formulations are presented in Figure 3. In the FTIR spectrum of the calcium alginate, a band was observed at 1630 cm^−1^ corresponding to the strong asymmetric stretching of the carboxylate, and another was observed around 1430 cm^−1^ produced by the weak symmetric stretching of –COO^−^. The strong and wide absorption band between 3600 and 3200 cm^−1^ was attributed to −OH stretching, characteristic of polysaccharides. The bending of −CH_2_ at 1028 cm^−1^ was also observed. The addition of bentonite has little influence on the position of the characteristic absorption peak of alginate. However, a decrease in the intensity of carboxylate stretching (1630 and 1430 cm^−1^) was observed, accompanied by an increase in the intensity of −CH_2_ bending around 1028 cm^−1^, which overlapped with the stretching of the Si–O of bentonite. 

The spectra presented in Figure 3 clearly show that the DDA molecules were successfully incorporated after all procedures. For all formulations containing DDA, the carbonyl band at 1744 cm^−1^, typical of ester compounds, was clearly detected in the alginate-bentonite-based formulations.

### 2.2. Laboratory Volatilization Studies

#### 2.2.1. Preliminary Volatilization Experiments

Preliminary release experiments were carried out to consider the influence that the alginate/bentonite ratio had on the DDA release rate.

**Figure 4:**
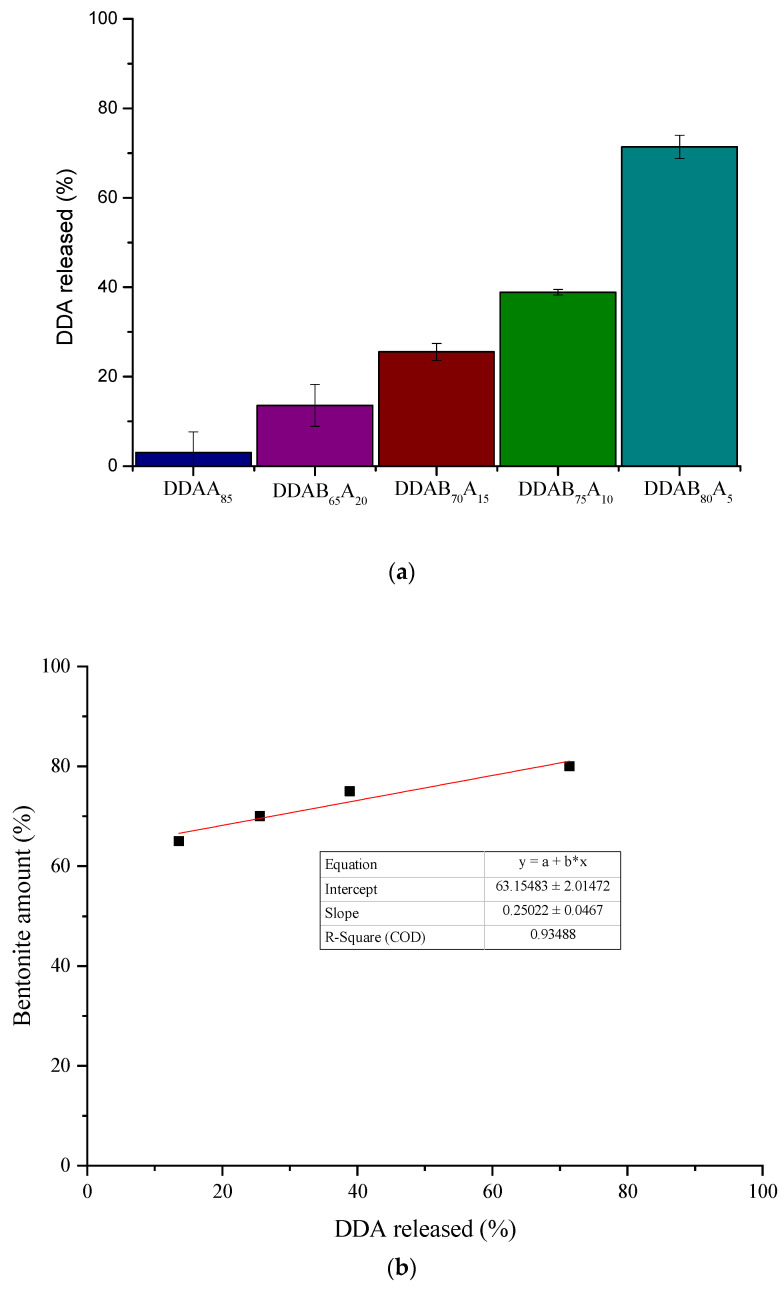
Percentage of DDA released from CRFs (**a**) and percentage of bentonite amount present in CRFs versus percentage of DDA released from CRFs (**b**).

The release-rate study was carried out over a period of 96 h in laboratory conditions (50 °C, airflow 120–130 L min^−1^) on DDA alginate and alginate/bentonite beads. Figure 4a shows the percentage of DDA released from the prepared formulations. After 4 days, the amount of active ingredient remaining in the granules ranged from 28.60% for DDAB_80_A_5_ to 96.98% for DDAA_85_. Regarding the release behavior of DDA, for the beads containing only alginate (DDAA_85_), the percentage of DDA released was 3.02%, which means that virtually the entire amount of encapsulated compound remained in the granules. These data showed that the alginate/bentonite blends can be used as slow-release devices. The release rate was highly influenced by the alginate/bentonite ratio, the amount of DDA released increasing as the alginate/bentonite ratio decreased. A linear relationship was found between the percentage of DDA released and the amount of bentonite present in each formulation (Figure 4b).

Based on the obtained results for the characterization parameters (EE, granule morphology, and thermal stability) of the polymeric networks and preliminary volatilization experiments, the DDAB_75_A_10_ formulation was selected as a model to study the release behavior of DDA from alginate/bentonite matrices under both laboratory and real-environment conditions (field experiments).

#### 2.2.2. Release Study

Considering that DDA evaporates under slightly harsh conditions (i.e., 50 °C and continuous airflow), we assumed that the selected alginate-bentonite formulation could also be used to regulate pheromone release for a long time under conditions close to actual temperature conditions. To test this, the release study was carried out at a temperature close to room temperature (25 °C).

**Figure 5:**
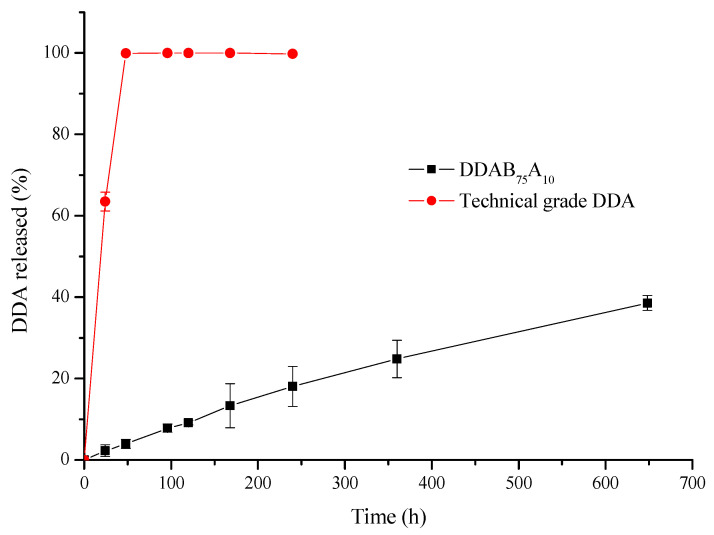
Cumulative release of DDA from the technical-grade product and the DDAB_75_A_10_ formulation in laboratory volatilization experiments.

The percentages of DDA released from the alginate/bentonite granules and the technical-grade product are shown in Figure 5. As is evident in Figure 5, the alginate/bentonite formulation (DDAB_75_A_10_) exhibited an extended active-ingredient release profile in contrast to the technical-grade product. For the technical-grade product, 100% of DDA was released in 48 h, while in the case of the formulation, after 648 h, only slightly more than 35% of the active ingredient had been released. 

For the DDAB_75_A_10_ formulation, a nearly linear relationship between the amount of DDA released and time was observed, indicating a constant release rate, which is highly desirable for controlled-release devices. This fact suggests that there may be a complex relationship between the chemistry and morphology of the granule surface (chemical affinity, surface area, porosity, etc.) and the observed release characteristics. 

To determine the most suitable release model to explain the kinetic profile, the DDA release curve was fitted using the mathematical models described in the Mathematical Modelling and Statistical Analysis section. The values of the release kinetic constants and the determination coefficients obtained are summarized in Table 4. 

There was a very good correlation of the release profiles of the DDA granules with the first-order and Ritger and Peppas equations, the determination coefficient (*R*^2^) being greater than 0.99 in both cases, and the worst was obtained with the Higuchi model.

In the Ritger and Peppas model, the values of n indicate the following release mechanisms: for *n* ≤ 0.43, the dominant release mechanism is Fickian diffusion (case I transport); 0.43 ≤ *n* < 0.85 indicates diffusion and a swelling release mechanism (non-Fickian or anomalous transport); and *n* ≥ 0.85 corresponds to zero-order release kinetics (case II transport) [30]. In the present study, for DDA release, the diffusion exponent was found to be equal to 0.818, based on which it was assumed that the diffusional release followed anomalous transport. However, this value is very close to the value corresponding to the zero-order release kinetics, indicating that diffusion is not the dominant mechanism of DDA release from the granules. The zero-order kinetic model is the most suitable to describe the pheromone release rate because it allows a constant release rate and avoids large emissions initially, which can minimize side effects.

Other authors have found similar behavior when studying the release of n-decanol from zeolitic matrices [31], Z (8)-dodecenyl acetate from paraffin wax [32], and pheromones with different functional groups from sol-gel formulations [33].

### 2.3. Field Volatilization Studies

**Figure 6:**
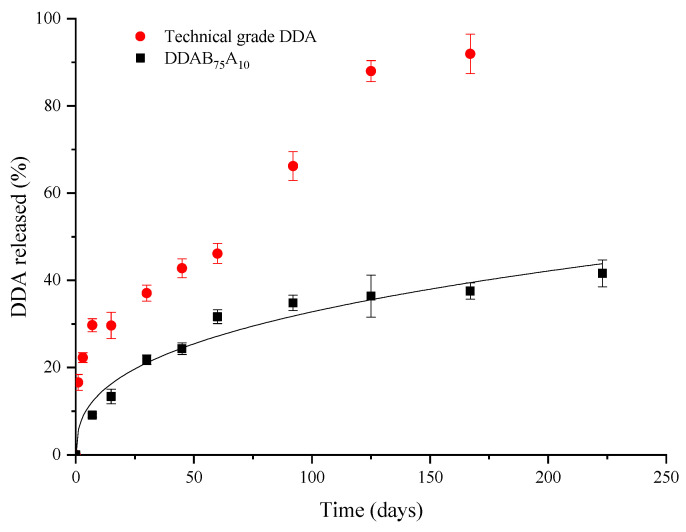
Cumulative release of DDA from the technical-grade product and the DDAB_75_A_10_ formulation in field experiments. Symbols are experimental data, and the line represents the Ritger and Peppas theoretical model.

The release patterns of DDA from CR granules and the volatilization of technical-grade DDA were followed throughout the experiment. In the case of the CRFs, the release of DDA was quantified by analyzing the granules recovered from the PVC dispensers on each sampling date. For the technical product, the remaining DDA in the dispenser was extracted. In both, the amount of volatilized DDA was determined by the difference with respect to the initial amount tested. These data are shown in Figure 6.

As observed in Figure 6, nearly 30% of DDA was released in 7 days from technical-grade DDA, while at least 60 days was necessary to achieve a similar percentage release of DDA for the DDAB_75_A_10_ formulation. More than 90% of DDA was released at 167 days for the technical product, evidencing an emission kinetic that can approach linear behavior, which implies a constant release of DDA over time.

Furthermore, Figure 6 shows that the temporal pattern of release of the DDAB_75_A_10_ formulation follows the kinetic behavior expected for monolithic systems for controlled-release formulations, with a high initial release of the compound followed by an exponential decrease in the release rate [9,10,34]. The release experimental data were fitted to the Ritger and Peppas model, with a determination coefficient (*R*^2^) equal to 0.965. The diffusion exponent (*n*) was found to be equal to 0.365, based on which it was assumed that the dominant release mechanism is Fickian diffusion (case I transport).

The amount of DDA released in the field experiments corroborates our previous results obtained in laboratory experiments. Furthermore, considering the relationship found between the bentonite content in the formulation and the percentage of DDA released, we were able to select the most suitable formulation to optimize the use of these formulations in agriculture as biological control devices.

## 3. Conclusions

In conclusion, new controlled-release formulations of dodecyl-acetate based on different contents of alginate and bentonite were successfully prepared. A relationship between alginate content and CRF encapsulation efficiency was obtained, with EE increasing as the amount of alginate increased.

From the SEM characterization studies, an increase in the size and roughness of the CRFs was observed with increasing bentonite content. Similar behavior was observed for the thermal stability of the CRFs. DDA molecules were successfully incorporated into alginate CRFs, as inferred from FT-IR spectra.

Based on preliminary volatilization experiments, a linear relationship was found between the percentage of DDA released and the amount of bentonite present in alginate CRFs. The laboratory release study showed an immediate release of DDA from the technical-grade product (100% of DDA released in 48 h), while the selected alginate-bentonite formulation (DDAB_75_A_10_) showed a prolonged release profile for DDA. The Ritger and Peppas model was suggested as the one most appropriate to describe the release kinetic profile of DDA from the selected formulation. From the value of the diffusional exponent obtained (*n* = 0.818), it can be inferred that the release process follows an anomalous or non-Fickian transport mechanism.

The results of the field volatilization studies, together with those obtained from the laboratory release experiments, allowed us to obtain a set of parameters that can be used to improve the release patterns of volatile compounds, such as DDA. Therefore, these new alginate-bentonite formulations could be incorporated as devices in agricultural biological control programs.

## 4. Materials and Methods

### 4.1. Materials

Technical-grade dodecyl acetate (DDA) (97%) was supplied by Merck Life Science S.L. (Madrid, Spain). Its molecular formula and selected properties are as follows: molecular formula, C_14_H_28_O_2_; molecular weight, 228.37 g mol^−1^; melting point, 150 °C at 15 mmHg; octanol/water partitioning (log K_ow_), 5.88.

Commercially available bentonite obtained from Merck Life Science S.L. (Madrid, Spain) was used in this study.

The solvent used in the DDA extraction was HPLC-grade hexane obtained from Merck Life Science S.L. (Madrid, Spain). Sodium alginate (medium viscosity, 3.5 kg m^−1^ s^−1^ for 2% solution) and tripolyphosphate (90–95%) obtained from Merck Life Science S.L. (Madrid, Spain) and calcium chloride (95%) obtained from Panreac S.A. (Barcelona, Spain) were used in the preparation and evaluation of the CR formulations.

### 4.2. Preparation of the Controlled-Release Formulations (CRFs)

The gelling properties of alginate in the presence of divalent cations were used as the main factors to prepare the CRFs. They were composed of formulations in water containing different percentages of dodecyl acetate (DDA), sodium alginate (A), and bentonite (B) (shown in Table 1). These mixtures were strongly shaken for 1 h. The pH values measured for these dispersions ranged between 7.5 and 8.2.

A 250 mL gellant bath of 0.25 M CaCl_2_ solution was used to dropwise add the mixtures (100 g) using the method described by Flores-Cespedes et al. [34]. The obtained granules were kept in the gellant bath for 20 min. Then, the beads were filtered and dried at room temperature for 72 h. The CR granules were kept in a desiccator with silica gel at room temperature before the experiments were carried out. The obtained CRFs are designated in the text as DDAA_85_, DDAB_65_A_20_, DDAB_70_A_15_, DDAB_75_A_10_, and DDAB_80_A_5_. The numbers in subscript indicate the percentage of each component in dry granules.

### 4.3. Granule Characterizations

#### 4.3.1. DDA Content in CRFs

The DDA concentration in the CRF granules was determined by breaking 20 mg of the granules into 5 mL of tripolyphosphate solution (0.03 M). To achieve the complete disintegration of the granules, an ultrasound bath was used for 15 min. Then, 80 mL of hexane was added for DDA extraction, and the conical flasks containing this solution were shaken for 24 h in a thermostatic bath at 25 °C ± 1 °C. The volume was made up to 100 mL with hexane. Nylon filters (0.20 µm) were used to filter the resulting extract. The concentration of DDA in the solution was determined by gas chromatography (GC) using a Thermo Fisher Scientific Focus GC System (Waltham, MA, USA) equipped with a split/splitless injector, a Thermo AS 3000 autosampler, an MS detector model ISQ, and an Xcalibur data station.

To analyze DDA, we used the conditions for GC analysis previously described by other authors [35,36]. These conditions were as follows: the separation was carried out on a 5 m × 0.25 mm I. D., 0.25 μm film thickness, model TG-5MS column obtained from Thermo Fisher Scientific (Waltham, MA, USA). Temperature program: initial temperature at 80 °C, held for 1 min, and a ramp at 8 °C/min to 210 °C, held for 10 min. Injection temperature: 240 °C. Injection volume: 1 μL. Carrier gas: He at constant flow rate, 1.0 mL min^−1^. Detection: temperature MS transfer line, 250 °C; temperature ion source, 230 °C. The detector was run in electron impact (EI) ionization mode at 70 eV. An external standard calibration curve was used to calculate the amount of DDA in the samples. Two replicates were carried out for each sample.

Dodecyl acetate practical loading was defined as the mass percentage of encapsulated DDA relative to the dry weight of the granules (alginate polymer plus bentonite plus encapsulated DDA), while dodecyl acetate encapsulation efficiency was designated as the percentage in weight of DDA trapped in microcapsules with respect to what had been initially added to the system (theoretical DDA loading). The following equations were used to calculate the dodecyl acetate practical loading and encapsulation efficiency:

(1) $$\mathrm{Practical}\;\mathrm{DDA}\;\mathrm{loading}\;(\%)=\left(\frac{\mathrm{amount}\;\mathrm{of}\;\mathrm{DDA}\;\mathrm{encapsulated}\;\mathrm{in}\;\mathrm{CR}\;\mathrm{granules}}{dry\;weight\;\mathrm{of}\;\mathrm{CR}\;\mathrm{granules}}\right)\times 100$$

(2) $$\mathrm{Encapsulation}\;\mathrm{efficiency}\;(\%)=\left(\frac{\mathrm{Practical}\;\mathrm{DDA}\;\mathrm{loading}}{\mathrm{Theoretical}\;\mathrm{DDA}\;\mathrm{loading}}\right)\times 100$$

#### 4.3.2. Thermogravimetric Analysis (TGA-DTG)

A TGA Q50 thermogravimetric analyzer obtained from TA Instruments (New Castle, DE, USA) was used to carry out the thermal stability analysis of pheromone formulations. Experiments were carried out in an atmosphere of synthetic air at 10 °C min^−1^, heating the CRF granules (5.0 ± 0.1 mg) from 20 °C to 900 °C. Universal Analysis 2000 V4.0 software obtained from TA Instruments was used to analyze the TGA curves.

#### 4.3.3. Differential Scanning Calorimetry (DSC)

A DSC Q20 obtained from TA Instruments was used to obtain DSC scans of DDA formulations. The samples (6 mg) were preheated to 120 °C for water evaporation. The heat involved in the heating process (10 °C/min) from room temperature to 300 °C was plotted as a function of temperature. A steam of 20 mL min^−1^ Nitrogen was used as inert atmosphere. 

#### 4.3.4. Fourier Transform Infrared Measurements

DDA formulation Fourier transform infrared (FT-IR) spectra were determined with an FT-IR Raman Vertex 70 obtained from Bruker (Billerica, MA, USA). The FT-IR spectra of samples (5% (*w*/*w*) in KBr were recorded between 400 and 4000 cm^−1^ at a resolution of 4 cm^−1^.

#### 4.3.5. Scanning Electron Microscopy (SEM)

A Hitachi S-3500-N scanning electron microscope obtained from Hitachi Instruments Ltd. (Tokyo, Japan) was used to inspect the morphology and structure of the alginate-bentonite-based CRF surfaces. The granules were explored at the required magnification at room temperature. The beads were placed on a brass holder and sputtered with a thin coat of gold under vacuum conditions. The acceleration voltage used was 10 kV. A secondary electron detector was used to produce SEM image.

### 4.4. Laboratory Volatility Studies

DDA technical-grade samples and a DDAA_10_B_75_ formulation were used to carry out the studies on DDA volatility in lab. These studies were conducted with a Micro-Chamber/Thermal Extractor (µ-CTE) obtained from Markes International, Inc. (Cincinnati, OH, USA). For the DDAB_75_A_10_ formulation and the DDA technical product, a quantity of granules or solution, respectively, containing approximately 3–4 mg of dodecyl acetate was accurately weighed and placed in a stainless-steel sample pan (interior volume of 45 mL, 28 mm deep and 45 mm in diameter). Then, the micro-chambers were closed and left for 10–15 min to achieve the experimental temperature (50 °C) before running the experiments. A rotameter attached to the micro-chamber outlets was used to check the airflow rates (120–130 mL min^−1^).

Different time intervals were used to carry out the volatilization experiments (range between 24 and 650 h). The pans were removed from µ-CTE for each collection sample time. The same method used formerly to quantify DDA content in the formulations was used to obtain the DDA amount remaining in the DDAB_75_A_10_ formulation. For technical-grade DDA, 25 mL of hexane was added to the sample pans and introduced in an ultrasound bath at room temperature for 15 min. The solution volume was made up with hexane to an end volume of 50 mL. Dodecyl acetate content was quantified by GC-MS with the same setup as described above. The experiments were conducted in triplicate.

### 4.5. Field Volatilization Studies

**Figure 7:**
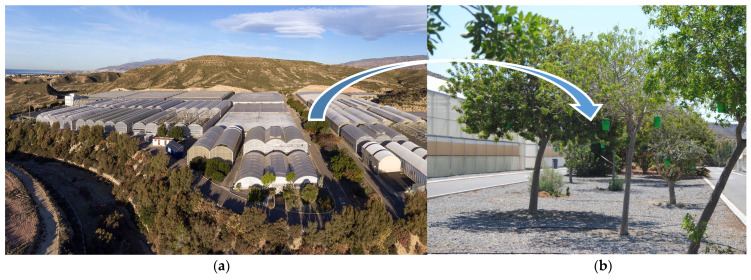
Experimental farm of the UAL-ANECOOP Foundation (**a**) and the plot where the containers of the samples for the field experiments were located (**b**).

The field experiment was carried out at the Experimental Farm of the UAL-ANECOOP Foundation, located in the area “Los Goterones” belonging to the town of Retamar, polygon 24, plot 281, in the municipality of Almería, Spain (Figure 7a). The geographical coordinates are: longitude, 2.1708° W; latitude, 36.5177° N.

The collection system dedicated to measuring the release of volatile compounds consisted of 0.5 L rigid plastic containers (FUNNEL PV, Semiotrap, (Jaén, Spain) containing the technical-grade DDA or DDAB_75_A_10_ formulations.

The containers with the samples were hung from the trees located in one of the plots on the farm (Figure 7b) at a height between 1.5 and 2.5 m from the ground.

The experiment was launched in June 2020, and evaluations were conducted for six months. At different time intervals, six containers were randomly selected, three containing the DDAB_75_A_10_ formulation and three containing the technical product. Then, in the laboratory, the amount of DDA was determined using the procedure described in Section 4.3.1.

### 4.6. Mathematical Modelling and Statical Analysis

To determine and interpret the release kinetics of DDA from the encapsulated granules, the release profiles were fitted to four different mathematical models of release of bioactive materials. The mathematical models of controlled release used were zero-order (3), first-order (4), Higuchi (5), and Ritger and Peppas (6) [30]:

(3) $$\mathrm{Zero}-\mathrm{order}\;\mathrm{kinetic}\;\mathrm{model}:\;\frac{M_{t}}{M_{0}}=k_{0}t+C$$

(4) $$\mathrm{First}-\mathrm{order}\;\mathrm{kinetic}\;\mathrm{model}:\;\frac{M_{t}}{M_{0}}=1-e^{-k_{1}t}$$

(5) $$\mathrm{Higuchi}\;\mathrm{kinetic}\;\mathrm{model}:\;\frac{M_{t}}{M_{0}}=kt^{1/2}$$

(6) $$\mathrm{Ritger}\;\mathrm{and}\;\mathrm{Peppas}\;\mathrm{model}:\;\frac{M_{t}}{M_{0}}=k_{p}t^{n}$$

where *t* is the release time, *M_t_* denotes the amount of DDA released measured at time *t*, *M*_0_ is the initial content of DDA in the granules, and therefore M_t_/M_0_ represents the fraction of DDA released at time t. *k*_0_, *k*_1_, *k_H_*, and *k_P_* are the kinetic release constants in the zero-order, first-order, Higuchi, and Ritger and Peppas models, respectively. The *n* value indicates the release exponent and therefore indicates the release mechanism of the active ingredient. The determination coefficients (*R*^2^) of these models were compared, and the model with the highest *R*^2^ was considered the most applicable to predict the release of DDA from DDAB_75_A_10_ granules.

The values of the release kinetic constants and the correlation coefficients were obtained by applying the models mentioned above to predict release rates, using the nonlinear curve-fitting utility of the Origin 7.0 software.

All experiments were performed in triplicate. The results were presented using the means and standard deviations (SDs) of three independently performed trials.

## Figures and Tables

**Table 1:** Percentages (by weight) of CRF components containing DDA.

Formulation	DDA (%)	Alginate (%)	Bentonite (%)	Water (%)
DDAA_85_	0.25	1.40	-	98.35
DDAB_65_A_20_	1.17	1.42	4.76	92.65
DDAB_70_A_15_	1.17	1.06	5.14	92.63
DDAB_75_A_10_	1.17	0.71	5.49	92.63
DDAB_80_A_5_	1.17	0.37	5.81	92.65

DDAA_85_: dodecyl acetate-alginate 85%; DDAB_65_A_20_: dodecyl acetate-bentonite 65% -alginate 20%; DDAB_70_A_15_: dodecyl acetate-bentonite 70% -alginate 15%; DDAB_75_A_10_: dodecyl acetate-bentonite 75% -alginate 10%; DDAB_80_A_5_: dodecyl acetate-bentonite 80% -alginate 5%.

**Table 2:** Characteristics of controlled-release formulations containing DDA. Values in parentheses represent the standard deviations.

Formulation	Theoretical DDA Loading ^a^ (%)	Practical DDA Loading (%)	Solids Recovery ^b^ (%)	Encapsulation Efficiency (%)
DDAA_85_	15.29	12.28 (0.84) ^c^	67.20	80.31
DDAB_65_A_20_	16.07	11.86 (0.15)	90.11	73.80
DDAB_70_A_15_	15.89	10.85 (0.75)	85.20	68.28
DDAB_75_A_10_	15.89	10.45 (1.25)	83.52	65.76
DDAB_80_A_5_	15.95	5.39 (0.28)	77.15	33.79

^a^ Theoretical DDA loading (%) = (weight of DDA used to prepare the formulations/total weight of solid components used in the formulation process) × 100; ^b^ Solids recovery (%) = (total weight of formulation recovered/total weight of solid components used in the formulation process) × 100; ^c^ Values in parentheses represent the standard deviations.

**Table 3:** Degradation temperatures and mass loss percentages of formulations containing DDA.

		DDAA_85_	DDAB_65_A_20_	DDAB_70_A_15_	DDAB_75_A_10_	DDAB_80_A_5_
Stage 1	Degradation temperature (°C)	20–150				
	Mass loss (%)	18.85	21.53	17.44	16.52	11.95
	DTG max. (°C)	49.00	43.78	40.42	43.11	40.42
Stage 2	Degradation temperature (°C)	150–350				
	Mass loss (%)	35.63	12.90	13.29	11.26	11.09
	DTG max. (°C)	220	272	269	237	280
Stage 3	Degradation temperature (°C)	350–550				
	Mass loss (%)	18.75	7.82	8.70	5.53	2.68
	DTG max. (°C)	500	479	503	515	472
Stage 4	Degradation temperature (°C)	550–750				
	Mass loss (%)	5.92	3.41		2.70	3.56
	DTG max. (°C)	678	593		589	567
T mass loss 50% (°C)		300				
Residue (800 °C)		19.84	54.49	59.48	63.57	70.36

**Table 4:** Constants and parameters obtained from the fitting of the DDA release data to the kinetic models for the DDAB_75_A_10_ formulation.

Model	Kinetic Constant	C	*n*	*R* ^2^
Zero-order	5.97 × 10^−4^	0.0179	-	0.986
First-order	7.8 × 10^−4^	-	-	0.996
Higuchi	0.012	-	-	0.890
Ritger and Peppas	19.5 × 10^−4^	-	0.818	0.997
